# Unusual Manifestations of Dengue Fever: A Review on Expanded Dengue Syndrome

**DOI:** 10.7759/cureus.10678

**Published:** 2020-09-27

**Authors:** Maheswaran Umakanth, Navaneethakrishnan Suganthan

**Affiliations:** 1 Clinical Medicine, Batticaloa Teaching Hospital, Colombo, LKA; 2 Internal Medicine, University Medical Unit Teaching Hospital Jaffna, Jaffna, LKA

**Keywords:** expanded dengue syndrome, dengue, plasma leakage

## Abstract

Dengue infection may manifest as dengue fever (DF), dengue hemorrhagic fever (DHF), and dengue shock syndrome (DSS). The World Health Organization (WHO) came up with the term "expanded dengue syndrome" (EDS) to designate cases which do not fall into either DHF or DSS, with unusual manifestations in other organs such as the cardiovascular system, the nervous system, the kidneys, the gut, and the hematological system, which have been increasingly reported and called EDS. Furthermore, EDS is becoming widespread globally with unusual features and increased severity. There are increasing reports of under-recognized and infrequent manifestations with severe organ involvement. This review gives knowledge of expanded dengue syndrome which helps to catch the diagnosis of dengue early, particularly during the ongoing epidemics and escaping from further series of unnecessary investigations.

## Introduction and background

Dengue is caused by a vector-borne arbovirus of the flavivirus family. It is transmitted by the female mosquitoes Aedes aegypti, Aedes albopictus, Aedes scutellaris, and Aedes polynesiensis. Dengue virus is an arbovirus disease-causing 390 million dengue infections per year, of which 96 million manifests clinically in nearly 129 countries around the world [1]. However, the burden of the disease is more evident in Asian countries. There has been a dramatic increase in the number of cases of dengue fever (DF) recently, with almost 50 million people infected every year [2]. Dengue is most commonly a self-limiting flu-like illness of low mortality, which can be asymptomatic. DF is characterized by abrupt onset of fever often accompanied by a severe headache and pain behind the eyes, muscle pain, joint pains, nausea, vomiting, abdominal pain, and loss of appetite. Two distinct clinical entities, dengue hemorrhagic fever (DHF) and dengue shock syndrome (DSS) have been associated with poorer outcomes. However, regardless of the stubborn grouping of dengue into DF, DHF, and DSS, overlap between the different manifestations could be observed [3]. 

Dengue is an acute and self-limited disease characterized by fever, headache, retro-orbital pain, myalgia, nausea, vomiting, skin rash, leukopenia, and thrombocytopenia associated with or without plasma leakage [4]. Furthermore, DSS can complicate with other organ involvement such as the liver, kidneys, heart, and brain. Apart from the common classical presentation, dengue infection can result in a spectrum of uncommon clinical manifestations, which are grouped under the term - expanded dengue syndrome (EDS) [5].

We performed a PubMed® and a Google Scholar search for all papers published in the English language using the keyword of expanded dengue syndrome and atypical manifestations of DF. All abstracts were read independently by the authors. Observational studies, case series, and fifteen case reports of patients with a confirmed diagnosis of dengue were screened for inclusion. In all included reports, laboratory diagnosis of dengue was considered essential by any of the established diagnostic tests such as positive dengue immunoglobulin M (IgM) antibody by the enzyme-linked immunosorbent assay (ELISA), or positive dengue NS1 antigen, or positive dengue polymerase chain reaction (PCR). We excluded reports where co-infection with other diseases was present. This review gives knowledge of EDS, which helps to clinch the diagnosis of dengue early, particularly during ongoing epidemics, escaping from further series of investigations.

## Review

Unusual manifestations

DF is characterized by abrupt onset of fever, often accompanied by a severe headache and pain behind the eyes, muscle pain, joint pains, nausea, vomiting, abdominal pain, and loss of appetite. Two distinct clinical entities, DHF and DSS, have been associated with poorer outcomes. Though, regardless of the dogged grouping of dengue into DF, DHF, and DSS, the overlap between the different manifestations could be observed. End organ damage, such as liver, kidney, heart, brain, and bone marrow involvement, has been mostly reported in severe dengue with plasma leakage, or bleeding, which may confuse the clinicians whether this end-organ damage is part of dengue shock or EDS.

EDS is a term announced by the WHO in 2011 to cover the uncommon expressions of dengue involving severe damage to the liver, kidneys, bone marrow, heart, or brain [6]. They may be related to underlying co-morbidities, associated co-infections, or prolonged shock. Certain high-risk groups such as expectant women, infants, elderly groups, and patients with coronary artery disease, hemoglobinopathies, and immunocompromised individuals are particularly susceptible to developing EDS. Clinicians must be aware of these atypical features so that they can suspect dengue early, especially during ongoing epidemics (Table 1).

**Table 1:** Unusual presentations of dengue infection

Involvement variations
Neurological involvement
Encephalopathy
Encephalitis
Intracranial hemorrhages
Mononeuropathies
polyneuropathies
Guillain-Barre Syndrome
Transverse myelitis
Cardiac involvement
Conduction abnormalities
Myocarditis
Pericarditis
Pulmonary involvement
Pleural effusion
Acute respiratory distress syndrome
Pulmonary hemorrhage
Pneumonitis
Liver and gut involvement
Hepatitis
Fulminant hepatic failure
Acalculous cholecystitis
Acute pancreatitis
Acute parotitis
Renal involvement
Acute renal failure
Proteinuria
immunoglobulin A nephropathy
Glomerulonephritis
Hemolytic uremic syndrome
Blood and bone involvement
Hemophagocytic lymphohistiocytosis
Idiopathic thrombocytopenic purpura
Disseminated intravascular coagulopathy
Hepatomegaly
Splenomegaly
Cytopenia
Lymphadenopathy

Pathogenesis of severe dengue fever

There have been several recent advances in understanding the pathogenesis of dengue; however, the precise mechanisms remain to be fully delineated. All four dengue virus serotypes (SE) have related to the spectrum of disease. However, certain genotypes of dengue virus (SE 2 and 3) have been shown to be more virulent and cause massive dengue epidemics [7]. Dengue SE-1 has been associated with the more severe disease during primary dengue infections [6]. However, dengue virus SE-4 has been shown to cause milder clinical disease. In fact, it was recently shown that patients with primary infection with dengue SE-1 had higher viral loads than those infected with other viral serotypes [8].

The typical of dengue cases are mild to moderate in severity; however, severe cases are mostly related to two recognized pathophysiologic trademarks, which are called plasma leakage and bleeding; it causes shock and death. Even though the underlying mechanisms for a severe form of dengue have not been fully explained, but the sturdy association of severe dengue with different serotypes and an immune-mediated reaction has been assumed. In severe dengue, both T-cell mediated and antibody-dependent mechanisms have been implicated [9]. It has been discovered that cross-reactive T cells and antibodies strongly involve the pathogenesis in secondary dengue infections [10]. Moreover, the pro-inflammatory cytokines, which result in the failure of endothelial function and high level of enhancing antibodies, have further augmented the pathogenesis of severe dengue [11-12].

Neurological manifestations

The spectrum of neurological complications has been reported in DF, which are ranging from encephalopathy, encephalitis, acute disseminated encephalomyelitis (ADEM), neuromyelitis-optica (NMO), optic neuritis, myelitis, encephalopathy, Guillain-Barré syndrome (GBS) to dengue associated stroke. The pathogenesis of neurological indices is associated with direct invasion of the central nervous system by the dengue virus, autoimmune reactions, and metabolic alterations [13]. Even though neurological manifestations are less common in dengue, encephalitis and encephalopathy are the most common neurological presentations of dengue infection. Encephalopathy is a clinical phenomenon in which a reduced level of awareness was observed, and analysis of spinal fluid is usually normal [14]. In addition to that, the autoimmune-mediated reaction may cause ADEM, NMO, optic neuritis, myelitis, encephalopathy, and GBS [14-15]. Moreover, Intracranial hemorrhage is one of the rare and life-threatening manifestations of the central nervous system involvement by dengue as a part of EDS, which is presented as dengue-associated stroke [16].

Gastrointestinal manifestations

Elevation of liver transaminases was greater in patients with DHF compared to DF, and aspartate transaminase (AST) levels were higher than alanine aminotransferase (ALT) levels. It is assumed that the latter may be a result of myositis [17]. Although the liver is frequently involved in dengue infection, hepatic injury is usually mild to moderate. However, there are reported cases of acute hepatic failure complicated with hepatic encephalopathy, hepatorenal syndrome, severe bleeding, and metabolic acidosis. It has also been reported that acute hepatic failure is known to occur in patients with dengue infection without plasma leakage [18]. The pathogenesis of liver involvement in dengue is yet unclear; direct viral infection of hepatocytes and Kupffer cells is hypothesized, although immune mechanisms are also believed to play a role [19]. Acalculous cholecystitis is a usually reported clinical and ultra-sonographic finding in dengue patients [20]. The precise mechanisms of acalculous cholecystitis are poorly explained. However, in DHF, the direct viral entry with leakage and effusion from the vascular compartment is believed to result in the thickening of the gallbladder. In addition, acute pancreatitis is a less commonly described manifestation in dengue [21]. The precise mechanism of acute pancreatitis in DHF is poorly understood; however, it is thought to result in direct virus invasion, causing inflammation and destruction of pancreatic acinar cells or dengue virus infection, triggering an autoimmune response to pancreatic cells [22].

Renal manifestations

Dengue has been related to various forms of renal involvement. These include electrolyte imbalance, acute kidney injury (AKI), proteinuria, glomerulonephritis, alanine aminotransferase (IgA) nephropathy, hemolytic uremic syndrome, and acute tubular necrosis [22]. The incidence of renal manifestations varies from 17% to 62% [23]. Hyponatremia is the most common electrolyte abnormality in DF. Various mechanisms have been postulated for decreased serum sodium levels, including temporary inappropriate antidiuretic hormone secretion and temporary failure of the sodium-potassium pump [24]. The mechanism for AKI is multifactorial, which includes direct viral action on renal tissue, hypoperfusion secondary to shock, and rhabdomyolysis [25]. Rhabdomyolysis has been reported in DF, which is one of the causes of AKI. It is associated with elevated serum creatine kinase and increased excretion of myoglobulin in the urine [26].

Cardiac manifestations

A diversity of cardiac manifestations has been reported in dengue infection. Cardiac involvement of varying degrees has been described ranging from arrhythmias to myocardial depression, pericarditis, and myocarditis [27]. The prevalence of myocarditis in DF ranges from 9% to 15% [27-29]. A variety of rhythm abnormalities were reported in DF, which ranges from sinus tachycardia, second-degree heart block, third-degree heart block, atrial fibrillation to paroxysmal supraventricular tachycardia [30].

Respiratory manifestations

The spectrum of pulmonary involvement can occur in DF, ranging from pleural effusion, pneumonitis, non-cardiogenic pulmonary edema to hemoptysis [31]. In DF, pleural effusion is the most frequent cause of dyspnea; usually, it is bilateral and seen in the context of plasma leakage syndrome [32]. Furthermore, small pleural effusion alone is not a reliable marker of plasma leakage, as it is a common finding in patients with non-dengue febrile illness and those with DF. Lung parenchymal involvements are less common, including ground glass abnormalities, the varying patterns of consolidation, interlobular septal thickening, and pulmonary hemorrhage [32]. 

Hematological manifestations

The foremost blood abnormalities are hematocrit, lymphocytosis, basophilia, monocytosis, thrombocytopenia, and atypical lymphocytosis. Disseminated intravascular coagulopathy (DIC), hemophagocytic lymphohistiocytosis (HLH), cytopenias, lymphadenopathy, hepatomegaly, and splenomegaly have been reported in dengue fever [33-34]. Furthermore, idiopathic thrombocytopenic purpura (ITP) and pancytopenia, with bone marrow biopsy revealing aplastic anemia, have been described following dengue fever [35].

## Conclusions

Dengue infection may manifest as an asymptomatic DF, DHF, and DSS. The WHO presented the term "expanded dengue syndrome" to designate cases that do not fall into either dengue shock syndrome or dengue hemorrhagic fever. The most common are cardiac and neurological manifestations and dengue encephalitis is a significant cause of the fatal outcome. The sensible knowledge about EDS helps to establish the diagnosis and prompt the appropriate treatment for dengue with unusual manifestations. Clinicians must be mindful of these unusual features so that they can suspect dengue early, especially during ongoing epidemics.
